# Supplementary material for: BLI-Based Functional Assay in Phage Display Benefits the Development of a PD-L1-Targeting Therapeutic Antibody
Source: Viruses. 2020 Jun 25;12(6):684. doi: 10.3390/v12060684 (PMC7354572; doi:10.3390/v12060684)
Supplement: Supplementary file 1 [file viruses-12-00684-s001.zip › Supplementary materials 300dp/Table S1.docx]

**Table S1.** List of primers for variable heavy and light chain gene amplification

| scFv clone No. | VHF | |
| --- | --- | --- |
| 1 | gcgGGTACCAGGATCCACTGGTCAGGTGCAGCTGGTAGAGTCT | |
| 2 | gcgGGTACCAGGATCCACTGGTCAGGTGCAGCTGGTAGAGTCT | |
| 3 | gcgGGTACCAGGATCCACTGGTCAGGTGCAGCTGGTGCAGTC | |
| 4 | gcgGGTACCAGGATCCACTGGTCAGGTGCAGCTGGTACAGTCT | |
| 5 | gcgGGTACCAGGATCCACTGGTCAGATGCAGCTGGTGCAGTC | |
| 6 | gcgGGTACCAGGATCCACTGGTCAGGTGCAGCTGGTGCAGTC | |
| 7 | gcgGGTACCAGGATCCACTGGTCAGGTGCAGCTGGTGCAGTC | |
| 8 | gcgGGTACCAGGATCCACTGGTCAGATGCAGCTGGTGCAGTC | |
| 9 | gcgGGTACCAGGATCCACTGGTCAGATGCAGCTGGTGGAGTCT | |
| 10 | gcgGGTACCAGGATCCACTGGTCAGGTGCAGCTGGTGGAGTC | |
| 11 | gcgGGTACCAGGATCCACTGGTCAGGTGCAGCTGGTGCAGTC | |
| 12 | | gcgGGTACCAGGATCCACTGGTCAGGTGCAGCTGGTGGAGTC |
| 13 | | gcgGGTACCAGGATCCACTGGTCAGATGCAGCTGGTGGAGTCT |
| 14 | | gcgGGTACCAGGATCCACTGGTCAGGTGCAGCTGGTGGAGTC |
| 15 | | gcgGGTACCAGGATCCACTGGTCAGGTGCAGCTGGTAGAGTCT |
| 16 | | gcgGGTACCAGGATCCACTGGTCAGATGCAGCTGGTGCAGTC |
| 17 | | gcgGGTACCAGGATCCACTGGTCAGGTGCAGCTGGTGCAGTC |
| 18 | | gcgGGTACCAGGATCCACTGGTCAGGTGCAGCTGGTGGAGTC |
| 21 | | gcgGGTACCAGGATCCACTGGTCAGGTGCAGCTGGTGGAGTC |
| 22 | | gcgGGTACCAGGATCCACTGGTCAGGTGCAGCTGGTACAGTCT |
| 23 | | gcgGGTACCAGGATCCACTGGTCAGGTGCAGCTGGTACAGTCT |
| 24 | | gcgGGTACCAGGATCCACTGGTCAGGTGCAGCTGGTGCAGTC |
| 25 | | gcgGGTACCAGGATCCACTGGTCAGGTGCAGCTGGTGGAGTC |
| 26 | | gcgGGTACCAGGATCCACTGGTCAGGTGCAGCTGGTGCAGTC |
| 27 | | gcgGGTACCAGGATCCACTGGTCAGATGCAGCTGGTGCAGTC |
| 28 | | gcgGGTACCAGGATCCACTGGTCAGGTGCAGCTGGTAGAGTCT |
| 29 | | gcgGGTACCAGGATCCACTGGTCAGGTGCAGCTGGTAGAGTCT |
| 30 | | gcgGGTACCAGGATCCACTGGTCAGGTGCAGCTGGTGGAGTC |
| 31 | | gcgGGTACCAGGATCCACTGGTCAGGTGCAGCTGGTGGAGTC |
| 32 | | gcgGGTACCAGGATCCACTGGTCAGGTGCAGCTGGTGCAGTC |
| 33 | | gcgGGTACCAGGATCCACTGGTCAGGTGCAGCTGGTGCAGTC |
| 34 | | gcgGGTACCAGGATCCACTGGTCAGGTGCAGCTGGTGGAGTC |
| 35 | | gcgGGTACCAGGATCCACTGGTCAGGTGCAGCTGGTACAGTCT |
| 36 | | gcgGGTACCAGGATCCACTGGTCAGATGCAGCTGGTGGAGTCG |
| 37 | | gcgGGTACCAGGATCCACTGGTCAGGTGCAGCTGGTACAGTCT |
| 38 | | gcgGGTACCAGGATCCACTGGTCAGATGCAGCTGGTGCAGTC |
| 39 | | gcgGGTACCAGGATCCACTGGTCAGGTGCAGCTGGTGCAGTC |
| 40 | | gcgGGTACCAGGATCCACTGGTCAGATGCAGCTGGTGGAGTCT |
| 41 | | gcgGGTACCAGGATCCACTGGTCAGGTGCAGCTGGTGGAGTC |
| 42 | | gcgGGTACCAGGATCCACTGGTCAGGTGCAGCTGGTAGAGTCG |
| 43 | | gcgGGTACCAGGATCCACTGGTCAGATGCAGCTGGTACAGTCTGG |
| 44 | | gcgGGTACCAGGATCCACTGGTCAGGTGCAGCTGGTGGAGTC |
| 45 | | gcgGGTACCAGGATCCACTGGTCAGGTGCAGCTGGTAGAGTCT |
| 46 | | gcgGGTACCAGGATCCACTGGTCAGGTGCAGCTGGTGGAGTC |
| 47 | | gcgGGTACCAGGATCCACTGGTCAGATGCAGCTGGTGCAGTC |
| 49 | | gcgGGTACCAGGATCCACTGGTCAGGTGCAGCTACAGGAGTCG |
| 50 | | gcgGGTACCAGGATCCACTGGTCAGGTGCAGCTGGTGGAGTC |
| 51 | | gcgGGTACCAGGATCCACTGGTCAGATGCAGCTGGTGCAGTC |
| 52 | | gcgGGTACCAGGATCCACTGGTCAGGTGCAGCTGGTGCAGTC |
| 53 | | gcgGGTACCAGGATCCACTGGTCAGATGCAGCTGGTAGAGTCTGG |
| 54 | | gcgGGTACCAGGATCCACTGGTCAGGTGCAGCTGGTGCAGTC |
| 55 | | gcgGGTACCAGGATCCACTGGTCAGGTGCAGCTGGTGGAGTC |
| 56 | | gcgGGTACCAGGATCCACTGGTCAGGTGCAGCTGGTAGAGTCT |
| 57 | | gcgGGTACCAGGATCCACTGGTCAGATGCAGCTGGTGCAGTC |
| 58 | | gcgGGTACCAGGATCCACTGGTCAGGTGCAGCTGGTGCAGTC |
| 59 | | gcgGGTACCAGGATCCACTGGTCAGATGCAGCTGGTGCAGTC |
| 60 | | gcgGGTACCAGGATCCACTGGTCAGATGCAGCTGGTGGAGTCT |
| 61 | | gcgGGTACCAGGATCCACTGGTCAGGTGCAGCTGGTGGAGTC |
| 62 | | gcgGGTACCAGGATCCACTGGTCAGGTGCAGCTGGTGCAGTC |
| 63 | | gcgGGTACCAGGATCCACTGGTCAGGTGCAGCTGGTACAGTCT |
| 64 | | gcgGGTACCAGGATCCACTGGTCAGATGCAGCTGGTACAGTCTGG |
| 65 | | gcgGGTACCAGGATCCACTGGTCAGGTGCAGCTGGTGGAGTC |
| 66 | | gcgGGTACCAGGATCCACTGGTCAGATGCAGCTGGTGGAGTCT |
| 67 | | gcgGGTACCAGGATCCACTGGTCAGGTGCAGCTGGTGCAGTC |
| 68 | | gcgGGTACCAGGATCCACTGGTCAGGTGCAGCTGGTGGAGTC |
| 69 | | gcgGGTACCAGGATCCACTGGTCAGGTGCAGCTGGTACAGTCT |
| 70 | | gcgGGTACCAGGATCCACTGGTCAGGTGCAGCTGGTGGAGTC |
| 71 | | gcgGGTACCAGGATCCACTGGTCAGGTGCAGCTGGTGCAGTC |
| 72 | | gcgGGTACCAGGATCCACTGGTCAGGTGCAGCTGGTACAGTCT |
| Enzyme | | KpnI |
|  | |  |
| scFv clone No. | | VHR |
| 1 | | cgatgggcccttggtgctagcTGAGGAGACGGTGACCATTG |
| 2 | | cgatgggcccttggtgctagcTGAGGAGACGGTGATCATTGTCC |
| 3 | | cgatgggcccttggtgctagcTGAGGAGACGGTGACCGTGG |
| 4 | | cgatgggcccttggtgctagcTGAGGAGACGGTGACCAGG |
| 5 | | cgatgggcccttggtgctagcTGAGGAGACGGTGACCAGacT |
| 6 | | cgatgggcccttggtgctagcTGAGGAGACGGTGACCGTGG |
| 7 | | cgatgggcccttggtgctagcTGAGGAGACGGTGACCGTGG |
| 8 | | cgatgggcccttggtgctagcTGAGGAGACGGTGACCGTGG |
| 9 | | cgatgggcccttggtgctagcTGAGGAGACGGTGACCCGG |
| 10 | | cgatgggcccttggtgctagcTGAGGAGACGGTGATCAGGG |
| 11 | | cgatgggcccttggtgctagcTGAGGAGACGGTGACCGTGG |
| 12 | | cgatgggcccttggtgctagcTGAGGAGACGGTGATCGTGGT |
| 13 | | cgatgggcccttggtgctagcTGAGGAGACGGTGACCATTG |
| 14 | | cgatgggcccttggtgctagcTGAGGAGACGGTGACCAGG |
| 15 | | cgatgggcccttggtgctagcTGAGGAGACGGTGACCGTGG |
| 16 | | cgatgggcccttggtgctagcTGAGGAGACGGTGACCGTGG |
| 17 | | cgatgggcccttggtgctagcTGAGGAGACGGTGACCGTGG |
| 18 | | cgatgggcccttggtgctagcTGAGGAGACGGTGACCGGG |
| 21 | | cgatgggcccttggtgctagcTGAGGAGACGGTGACCAGG |
| 22 | | cgatgggcccttggtgctagcTGAGGAGACGGTGACCAGG |
| 23 | | cgatgggcccttggtgctagcTGAGGAGACGGTGATCATTGTCC |
| 24 | | cgatgggcccttggtgctagcTGAGGAGACGGTGACCGTGG |
| 25 | | cgatgggcccttggtgctagcTGAGGAGACGGTGATCATTGTCC |
| 26 | | cgatgggcccttggtgctagcTGAGGAGACGGTGACCATTG |
| 27 | | cgatgggcccttggtgctagcTGAGGAGACGGTGATCACTGT |
| 28 | | cgatgggcccttggtgctagcTGAGGAGACGGTGACCGTGG |
| 29 | | cgatgggcccttggtgctagcTGAGGAGACGGTGATCATTGTCC |
| 30 | | cgatgggcccttggtgctagcTGAGGAGACGGTGATCAGGG |
| 31 | | cgatgggcccttggtgctagcTGAGGAGACGGTGACCAGG |
| 32 | | cgatgggcccttggtgctagcTGAGGAGACGGTGACCCGG |
| 33 | | cgatgggcccttggtgctagcTGAGGAGACGGTGACCGTGG |
| 34 | | cgatgggcccttggtgctagcTGAGGAGACGGTGACCATTG |
| 35 | | cgatgggcccttggtgctagcTGAGGAGACGGTGACCAGG |
| 36 | | cgatgggcccttggtgctagcTGAGGAGACGGTGATCATTGTCC |
| 37 | | cgatgggcccttggtgctagcTGAGGAGACGGTGACCAGG |
| 38 | | cgatgggcccttggtgctagcTGAGGAGACGGTGACCGCG |
| 39 | | cgatgggcccttggtgctagcTGAGGAGACGGTGATCGGG |
| 40 | | cgatgggcccttggtgctagcTGAGGAGACGGTGACCAGG |
| 41 | | cgatgggcccttggtgctagcTGAGGAGACGGTGACCGTGG |
| 42 | | cgatgggcccttggtgctagcTGAGGAGACGGTGATCAGGG |
| 43 | | cgatgggcccttggtgctagcTGAGGAGACGGTGATCATTGTTCC |
| 44 | | cgatgggcccttggtgctagcTGAGGAGACGGTGACCGTGG |
| 45 | | cgatgggcccttggtgctagcTGAGGAGACGGTGACCATGG |
| 46 | | cgatgggcccttggtgctagcTGAGGAGACGGTGATCATTGTCC |
| 47 | | cgatgggcccttggtgctagcTGAGGAGACGGTGACCAGG |
| 49 | | cgatgggcccttggtgctagcTGAGGAGACGGTGACCACTGT |
| 50 | | cgatgggcccttggtgctagcTGAGGAGACGGTGACCGTGG |
| 51 | | cgatgggcccttggtgctagcTGAGGAGACGGTGACCGTGG |
| 52 | | cgatgggcccttggtgctagcTGAGGAGACGGTGACCAGG |
| 53 | | cgatgggcccttggtgctagcTGAGGAGACGGTGACCGTAG |
| 54 | | cgatgggcccttggtgctagcTGAGGAGACGGTGACCGTTG |
| 55 | | cgatgggcccttggtgctagcTGAGGAGACGGTGACCATTG |
| 56 | | cgatgggcccttggtgctagcTGAGGAGACGGTGATCAGGG |
| 57 | | cgatgggcccttggtgctagcTGAGGAGACGGTGACCAGG |
| 58 | | cgatgggcccttggtgctagcTGAGGAGACGGTGACCACTGT |
| 59 | | cgatgggcccttggtgctagcTGAGGAGACGGTGACCATTG |
| 60 | | cgatgggcccttggtgctagcTGAGGAGACGGTGATCAGTGT |
| 61 | | cgatgggcccttggtgctagcTGAGGAGACGGTGACCAGG |
| 62 | | cgatgggcccttggtgctagcTGAGGAGACGGTGACCAATGT |
| 63 | | cgatgggcccttggtgctagcTGAGGAGACGGTGACCATTG |
| 64 | | cgatgggcccttggtgctagcTGAGGAGACGGTGACCATTG |
| 65 | | cgatgggcccttggtgctagcTGAGGAGACGGTGACCAGG |
| 66 | | cgatgggcccttggtgctagcTGAGGAGACGGTGACCAGG |
| 67 | | cgatgggcccttggtgctagcTGAGGAGACGGTGACCAAAGT |
| 68 | | cgatgggcccttggtgctagcTGAGGAGACGGTGACCAGG |
| 69 | | cgatgggcccttggtgctagcTGAGGAGACGGTGATCGTGGT |
| 70 | | cgatgggcccttggtgctagcTGAGGAGACGGTGACCAGG |
| 71 | | cgatgggcccttggtgctagcTGAGGAGACGGTGACCATTG |
| 72 | | cgatgggcccttggtgctagcTGAGGAGACGGTGACCATTG |
| Enzyme | | NheI |
|  | |  |
| scFv clone No. | | VLF |
| 1 | | gcgGGTACCAGGATCCACTGGTTGAGGAGACGGTGACCACTGT |
| 2 | | gcgGGTACCAGGATCCACTGGTGACATCCAGATGACCCAGTCTCC |
| 3 | | gcgGGTACCAGGATCCACTGGTGACATCCAGATGACCCAGTCTCC |
| 4 | | gcgGGTACCAGGATCCACTGGTCAGCTCGTGCTGACTCAGCC |
| 5 | | gcgGGTACCAGGATCCACTGGTGACATCCAGATGACCCAGTCTCC |
| 6 | | gcgGGTACCAGGATCCACTGGTGACATCCAGATGACCCAGTCTCC |
| 7 | | gcgGGTACCAGGATCCACTGGTGACATCCAGATGACCCAGTCTCC |
| 8 | | gcgGGTACCAGGATCCACTGGTCAGTCTGCCCTGACTCAGCC |
| 9 | | gcgGGTACCAGGATCCACTGGTTCCTATGAGCTGACACAGGCA |
| 10 | | gcgGGTACCAGGATCCACTGGTGACATCCAGATGACCCAGTCTCC |
| 11 | | gcgGGTACCAGGATCCACTGGTGACATCCAGATGACCCAGTCTCC |
| 12 | | gcgGGTACCAGGATCCACTGGTGACATCCAGATGACCCAGTCTCC |
| 13 | | gcgGGTACCAGGATCCACTGGTTCCTATGAGCTGACACAGGCA |
| 14 | | gcgGGTACCAGGATCCACTGGTTCCTATGAGCTGACTCAGCCA |
| 15 | | gcgGGTACCAGGATCCACTGGTGACATCCAGATGACCCAGTCTCC |
| 16 | | gcgGGTACCAGGATCCACTGGTGACATCCAGATGACCCAGTCTCC |
| 17 | | gcgGGTACCAGGATCCACTGGTGACATCCAGATGACCCAGTCTCC |
| 18 | | gcgGGTACCAGGATCCACTGGTGACATCCAGATGACCCAGTCTCC |
| 21 | | gcgGGTACCAGGATCCACTGGTCAGCTCGTGCTGACTCAGCC |
| 22 | | gcgGGTACCAGGATCCACTGGTGACATCCAGATGACCCAGTCTCC |
| 23 | | gcgGGTACCAGGATCCACTGGTGACATCCAGATGACCCAGTCTCC |
| 24 | | gcgGGTACCAGGATCCACTGGTCAGCTCGTGCTGACTCAGCC |
| 25 | | gcgGGTACCAGGATCCACTGGTGACATCCAGATGACCCAGTCTCC |
| 26 | | gcgGGTACCAGGATCCACTGGTGACATCCAGATGACCCAGTCTCC |
| 27 | | gcgGGTACCAGGATCCACTGGTTCCTATGAGCTGACACAGCCA |
| 28 | | gcgGGTACCAGGATCCACTGGTGACATCCAGATGACCCAGTCTCC |
| 29 | | gcgGGTACCAGGATCCACTGGTTCCTATGAGCTGACACAGGCA |
| 30 | | gcgGGTACCAGGATCCACTGGTTCCTATGAGCTGACACAGCCA |
| 31 | | gcgGGTACCAGGATCCACTGGTGACATCCAGATGACCCAGTCTCC |
| 32 | | gcgGGTACCAGGATCCACTGGTAATTTTATGCTGACTCAGCCCCC |
| 33 | | gcgGGTACCAGGATCCACTGGTCAGTCTGCCCTGACTCAGCC |
| 34 | | gcgGGTACCAGGATCCACTGGTGATATTGTGATGACCCAGACTCCA |
| 35 | | gcgGGTACCAGGATCCACTGGTAATTTTATGCTGACTCAGCCCCAC |
| 36 | | gcgGGTACCAGGATCCACTGGTGACATCCAGATGACCCAGTCTCC |
| 37 | | gcgGGTACCAGGATCCACTGGTCAGCTCGTGCTGACTCAGCC |
| 38 | | gcgGGTACCAGGATCCACTGGTGACATCCAGATGACCCAGTCTCC |
| 39 | | gcgGGTACCAGGATCCACTGGTAATTTTATGCTGACTCAGCCCCC |
| 40 | | gcgGGTACCAGGATCCACTGGTCAGTCTGCCCTGACTCAGCC |
| 41 | | gcgGGTACCAGGATCCACTGGTGACATCCAGATGACCCAGTCTCC |
| 42 | | gcgGGTACCAGGATCCACTGGTCAGTCTGCCCTGACTCAGCC |
| 43 | | gcgGGTACCAGGATCCACTGGTTCCTATGAGCTGACACAGCCA |
| 44 | | gcgGGTACCAGGATCCACTGGTTCCTATGAGCTGACTCAGGCA |
| 45 | | gcgGGTACCAGGATCCACTGGTAATTTTATGCTGACTCAGCCCCAC |
| 46 | | gcgGGTACCAGGATCCACTGGTGACATCCAGATGACCCAGTCTCC |
| 47 | | gcgGGTACCAGGATCCACTGGTGACATCCAGATGACCCAGTCTCC |
| 49 | | gcgGGTACCAGGATCCACTGGTGACATCCAGATGACCCAGTCTCC |
| 50 | | gcgGGTACCAGGATCCACTGGTCAGTCTGCCCTGACTCAGCC |
| 51 | | gcgGGTACCAGGATCCACTGGTGACATCCAGATGACCCAGTCTCC |
| 52 | | gcgGGTACCAGGATCCACTGGTGACATCCAGATGACCCAGTCTCC |
| 53 | | gcgGGTACCAGGATCCACTGGTGACATCCAGATGACCCAGTCTCC |
| 54 | | gcgGGTACCAGGATCCACTGGTGACATCCAGATGACCCAGTCTCC |
| 55 | | gcgGGTACCAGGATCCACTGGTCAGCTCGTGCTGACTCAGCC |
| 56 | | gcgAGATCTACTGGTTCCTATGAGCTGACTCAGCCA |
| 57 | | gcgGGTACCAGGATCCACTGGTTCCTATGAGCTGACACAGCCA |
| 58 | | gcgGGTACCAGGATCCACTGGTCAGTCTGCCCTGACTCAGCC |
| 59 | | gcgGGTACCAGGATCCACTGGTGACATCCAGATGACCCAGTCTCC |
| 60 | | gcgGGTACCAGGATCCACTGGTGACATCCAGATGACCCAGTCTCC |
| 61 | | gcgGGTACCAGGATCCACTGGTAATTTTATGCTGACTCAGCCCCAC |
| 62 | | gcgGGTACCAGGATCCACTGGTGACATCCAGATGACCCAGTCTCC |
| 63 | | gcgGGTACCAGGATCCACTGGTTCCTATGAGCTGACACAGCCA |
| 64 | | gcgGGTACCAGGATCCACTGGTAATTTTATGCTGACTCAGCCCCAC |
| 65 | | gcgGGTACCAGGATCCACTGGTTCCTATGAGCTGACACAGCCA |
| 66 | | gcgGGTACCAGGATCCACTGGTTCCTATGAGCTGACTCAGGCA |
| 67 | | gcgAGATCTACTGGTTCCTATGAGCTGACTCAGGCA |
| 68 | | gcgAGATCTACTGGTTCCTATGAGCTGACTCAGGCA |
| 69 | | gcgAGATCTACTGGTTCCTATGAGCTGACACAGCCA |
| 70 | | gcgGGTACCAGGATCCACTGGTCAGCTCGTGCTGACTCAGCC |
| 71 | | gcgGGTACCAGGATCCACTGGTGACATCCAGATGACCCAGTCTCC |
| 72 | | gcgGGTACCAGGATCCACTGGTAATTTTATGCTGACTCAGCCCCAC |
| Enzyme | | BamHI & BglII (clones 56, 67, 68 & 69 ) |
|  | |  |
| scFv clone No. | | VLR |
| 1 | | gcccgtacgAATTTTATGCTGACTCAGCCCCAC |
| 2 | | gcccgtacgTTTGATCTCCACCTTGGTCCCT |
| 3 | | gcccgtacgTTTGATATCCACCTTGGTCCCTC |
| 4 | | gcccgtacgTAGGACGGTGACCTTGGTCC |
| 5 | | gcccgtacgTTTGATTTCCACCTTGGTCCCTC |
| 6 | | gcccgtacgTTTGATCTCCAGCTTGGTCCCC |
| 7 | | gcccgtacgTTTGATCTCCAGCTTGGTCCCC |
| 8 | | gcccgtacgTAGGACGGTCAGCTTGGTCC |
| 9 | | gcccgtacgTAGGACGGTCAGCTTGGTCC |
| 10 | | gcccgtacgTTTGATTTCCAGTCGTGTCCCTTG |
| 11 | | gcccgtacgTTTGATTTCCACCTTGGTCCCTC |
| 12 | | gcccgtacgTTTGATTTCCAGCTTGGTCCCTC |
| 13 | | gcccgtacgTAGGACGGTCAGCTTGGTCC |
| 14 | | gcccgtacgTAGGACGGTGACCTTGGTCC |
| 15 | | gcccgtacgTTTGATTTCCAGTCGTGTCCCTTG |
| 16 | | gcccgtacgTTTGATATCCACCTTGGTCCCTC |
| 17 | | gcccgtacgTTTGATTTCCACCTTGGTCCCTT |
| 18 | | gcccgtacgTTTGATCTCTACCTTGGTCCCTCC |
| 21 | | gcccgtacgTAGGACGGTGACCTTGGTCC |
| 22 | | gcccgtacgTTTGATCTCCAGTCGTGTCCCT |
| 23 | | gcccgtacgTTTGATATCCAGTCGTGTCCCTTG |
| 24 | | gcccgtacgTAGGACGGTCAGCTTGGTCC |
| 25 | | gcccgtacgTTTGATATCCACTTTGGTCCCAGG |
| 26 | | gcccgtacgTTTGATATCCACCTTGGTCCCTTG |
| 27 | | gcccgtacgTAGGACGGTGACCTTGGTCC |
| 28 | | gcccgtacgTTTGATCTCTAGCTTGGTCCCCT |
| 29 | | gcccgtacgTAGGACGGTCAGCTTGGTCC |
| 30 | | gcccgtacgTAGGACGGTGACTTTGGTCCC |
| 31 | | gcccgtacgTTTGATTTCCAGTCGTGTCCCTTG |
| 32 | | gcccgtacgTAGGACGGTGACCTTGGTCC |
| 33 | | gcccgtacgTAGGACGGTCAGCTTGGTCC |
| 34 | | gcccgtacgTTTGATATCCACTTTGGTCCCAGG |
| 35 | | gcccgtacgTAGGACGGTCAGCTTGGTCC |
| 36 | | gcccgtacgTTTGATTTCCAGCTTGGTCCCC |
| 37 | | gcccgtacgTAGGACGGTGACCTTGGTCC |
| 38 | | gcccgtacgTTTGATATCCACCTTGGTCCCTTG |
| 39 | | gcccgtacgTAGGACGGTGACCTTGGTCC |
| 40 | | gcccgtacgTAGGACGGTGACCTTGGTCC |
| 41 | | gcccgtacgTTTGATTTCCACCTTGGTCCCTC |
| 42 | | gcccgtacgTAGGACGGTGACCTTGGTCC |
| 43 | | gcccgtacgTAGGACGGTCAGCTTGGTCC |
| 44 | | gcccgtacgTAGGACGGTCAGCTTGGTCC |
| 45 | | gcccgtacgTAGGACGGTCAGCTTGGTCC |
| 46 | | gcccgtacgTTTGATATCCACCTTGGTCCCTTG |
| 47 | | gcccgtacgTTTGATCTCCACCTTGGTCCCT |
| 49 | | gcccgtacgTTTGATTTCCACCTTGGTCCCTC |
| 50 | | gcccgtacgTAGGACGGTCAGCTTGGTCC |
| 51 | | gcccgtacgTTTGATATCCACCTTGGTCCCTC |
| 52 | | gcccgtacgTTTGATCTCCACCTTGGTCCCT |
| 53 | | gcccgtacgTTTGATTTCCACCTTGGTCCCTC |
| 54 | | gcccgtacgTTTGATCTCCAGTCGTGTCCCT |
| 55 | | gcccgtacgTAGGACGGTGACCTTGGTCC |
| 56 | | gcccgtacgTAGGACGGTGACCTTGGTCC |
| 57 | | gcccgtacgTAGGACGGTGACCTTGGTCC |
| 58 | | gcccgtacgTAGGACGGTCAGCTTGGTCC |
| 59 | | gcccgtacgTTTGATCTCCACCTTGGTCCCT |
| 60 | | gcccgtacgTTTGATATCCACCTTGGTCCCTC |
| 61 | | gcccgtacgTAGGACGGTCAGCTTGGTCC |
| 62 | | gcccgtacgTTTGATATCTACTTTGGTCCCAGGG |
| 63 | | gcccgtacgTAGGACGGTCAGCTTGGTCC |
| 64 | | gcccgtacgTAGGACGGTCAGCTTGGTCC |
| 65 | | gcccgtacgTAGGACGGTCAGCTTGGTCC |
| 66 | | gcccgtacgTAGGACGGTCAGCTTGGTCC |
| 67 | | gcccgtacgTAGGACGGTCAGCTTGGTCC |
| 68 | | gcccgtacgTAGGACGGTCAGCTTGGTCC |
| 69 | | gcccgtacgTAGGACGGTGACCTTGGTCC |
| 70 | | gcccgtacgTAGGACGGTCAGCTTGGTCC |
| 71 | | gcccgtacgTTTGATTTCCAGTCGTGTCCCTTG |
| 72 | | gcccgtacgTAGGACGGTCAGCTTGGTCC |
| Enzyme | | BsiWI |
