# Supplementary material for: BLI-Based Functional Assay in Phage Display Benefits the Development of a PD-L1-Targeting Therapeutic Antibody
Source: Viruses. 2020 Jun 25;12(6):684. doi: 10.3390/v12060684 (PMC7354572; doi:10.3390/v12060684)
Supplement: Supplementary file 1 [file viruses-12-00684-s001.zip › Supplementary materials 300dp/Table S2.docx]

**Table S2.** Enrichment of phage from each round of panning.

| Round | Input (CFU)^1^ | Output (CFU) | Ratio (Output/input) |
| --- | --- | --- | --- |
| Round 1 | 1.00 x 10^13^ | 2.30 x 10^9^ | 2.30 x 10^-4^ |
| Round 2 | 5.60 x 10^12^ | 1.00 x 10^8^ | 1.79 x 10^-5^ |
| Round 3 | 1.50 x 10^13^ | 4.30 x 10^7^ | 2.87 x 10^-6^ |
| Round 4 | 1.50 x 10^13^ | 1.20 x 10^9^ | 8.00 x 10^-5^ |

^1^ CFU (colony-forming units)
