# Supplementary material for: BLI-Based Functional Assay in Phage Display Benefits the Development of a PD-L1-Targeting Therapeutic Antibody
Source: Viruses. 2020 Jun 25;12(6):684. doi: 10.3390/v12060684 (PMC7354572; doi:10.3390/v12060684)
Supplement: Supplementary file 1 [file viruses-12-00684-s001.zip › Supplementary materials 300dp/Table S3.docx]

**Table S3.** Characterization of 72 scFv antibodies for primary screening after phage panning.

| **scFv clone #** | **BLI-based assay (Inhibition %)** | **FACS (MFI)** | **ELISA**  **(450 nm)** | **IgG**  **expression** |
| --- | --- | --- | --- | --- |
|  |  |  |  |  |
| 1 | -6.07 | 45.40 | 0.228 |  |
| 2 | 6.88 | 63.70 | 1.256 | ■ |
| 3 | 18.61 | 84.50 | 2.244 | ■ |
| 4 | 11.05 | 38.70 | 0.383 |  |
| 5 | 8.77 | 72.40 | 0.477 |  |
| 6 | 18.19 | 43.30 | 0.988 | ■ |
| 7 | 46.55 | 126.00 | 1.572 |  |
| 8 | 51.70 | 49.40 | 1.397 | ■ |
| 9 | -7.08 | 37.30 | 0.961 |  |
| 10 | -13.68 | 42.80 | 0.665 | ■ |
| 11 | 37.92 | 32.50 | 0.724 | ■ |
| 12 | 8.74 | 38.60 | 0.542 |  |
| 13 | 27.55 | 33.40 | 0.355 | ■ |
| 14 | 16.20 | 27.70 | 0.64 | ■ |
| 15 | 8.74 | 35.40 | 1.127 | ■ |
| 16 | 7.71 | 30.40 | 0.961 |  |
| 17 | -4.92 | 40.70 | 1.904 |  |
| 18 | 23.82 | 43.90 | 0.417 | ■ |
| 19 | 25.27 | 37.20 | 0.416 |  |
| 20 | 20.59 | 34.00 | 0.843 | ■ |
| 21 | 8.53 | 44.40 | 0.146 |  |
| 22 | 32.98 | 34.40 | 1.394 | ■ |
| 23 | 5.06 | 22.40 | 0.12 |  |
| 24 | 45.79 | 21.60 | 0.483 | ■ |
| 25 | -2.84 | 32.70 | 0.362 |  |
| 26 | 34.37 | 26.10 | 1.685 | ■ |
| 27 | 63.70 | 21.10 | 0.668 | ■ |
| 28 | 25.13 | 25.70 | 1.088 |  |
| 29 | 11.30 | 24.10 | 0.178 | ■ |
| 30 | 7.07 | 26.30 | 0.157 |  |
| 31 | 6.25 | 73.30 | 1.775 |  |
| 32 | 13.32 | 25.50 | 0.349 | ■ |
| 33 | 2.78 | 28.20 | 0.351 |  |
| 34 | 6.83 | 95.70 | 0.135 |  |
| 35 | 51.84 | 39.20 | 1.688 | ■ |
| 36 | 22.78 | 88.80 | 1.938 | ■ |
| 37 | 6.34 | 24.10 | 0.13 |  |
| 38 | -0.05 | 27.70 | 1.286 |  |
| 39 | 2.22 | 27.00 | 0.301 |  |
| 40 | 13.16 | 124.00 | 1.592 | ■ |
| 41 | 10.45 | 20.20 | 1.994 | ■ |
| 42 | 0.81 | 48.80 | 0.463 |  |
| 43 | 21.15 | 84.30 | 0.691 |  |
| 44 | 28.21 | 30.60 | 0.329 | ■ |
| 45 | 24.49 | 37.30 | 0.472 | ■ |
| 46 | 19.04 | 56.10 | 0.897 |  |
| 47 | 12.80 | 39.30 | 0.428 | ■ |
| 48 | 24.16 | 57.80 | 0.983 |  |
| 49 | 13.96 | 43.80 | 0.985 | ■ |
| 50 | 17.43 | 157.00 | 1.572 | ■ |
| 51 | 21.95 | 54.90 | 0.467 | ■ |
| 52 | 10.78 | 51.70 | 0.777 | ■ |
| 53 | 4.51 | 63.50 | 0.431 |  |
| 54 | 6.41 | 43.70 | 1.558 |  |
| 55 | 21.74 | 32.00 | 0.589 | ■ |
| 56 | 9.29 | 29.90 | 0.148 | ■ |
| 57 | 12.79 | 69.30 | 0.13 | ■ |
| 58 | 25.10 | 86.60 | 0.409 | ■ |
| 59 | 28.19 | 35.60 | 1.397 | ■ |
| 60 | 9.34 | 141.00 | 0.892 | ■ |
| 61 | 17.17 | 34.90 | 0.298 | ■ |
| 62 | -0.83 | 30.80 | 0.14 |  |
| 63 | 18.00 | 87.30 | 1.79 | ■ |
| 64 | 9.94 | 142.00 | 1.611 |  |
| 65 | 5.70 | 52.90 | 0.222 |  |
| 66 | 7.57 | 39.50 | 0.245 |  |
| 67 | 35.74 | 94.10 | 1.319 | ■ |
| 68 | 25.16 | 22.50 | 0.19 |  |
| 69 | 14.81 | 24.90 | 0.751 | ■ |
| 70 | 24.39 | 49.80 | 0.699 | ■ |
| 71 | -1.78 | 28.00 | 0.408 | ■ |
| 72 | 5.30 | 136.00 | 1.921 |  |
