# Supplementary figures and images for: BLI-Based Functional Assay in Phage Display Benefits the Development of a PD-L1-Targeting Therapeutic Antibody
Source: Viruses. 2020 Jun 25;12(6):684. doi: 10.3390/v12060684 (PMC7354572; doi:10.3390/v12060684)

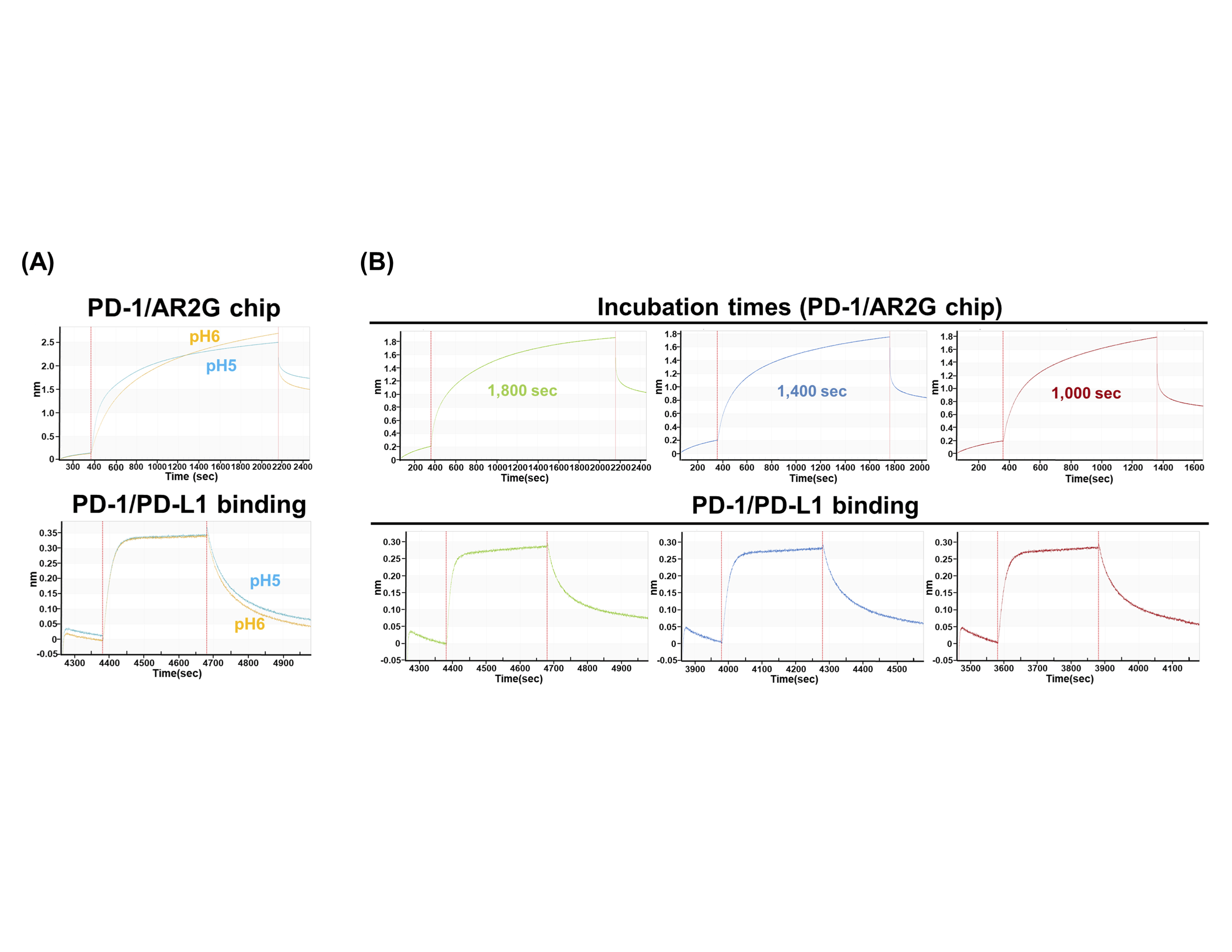

Supplement: Supplementary file 1 [file viruses-12-00684-s001.zip › Supplementary materials 300dp/Figure S1_rev2.tif]

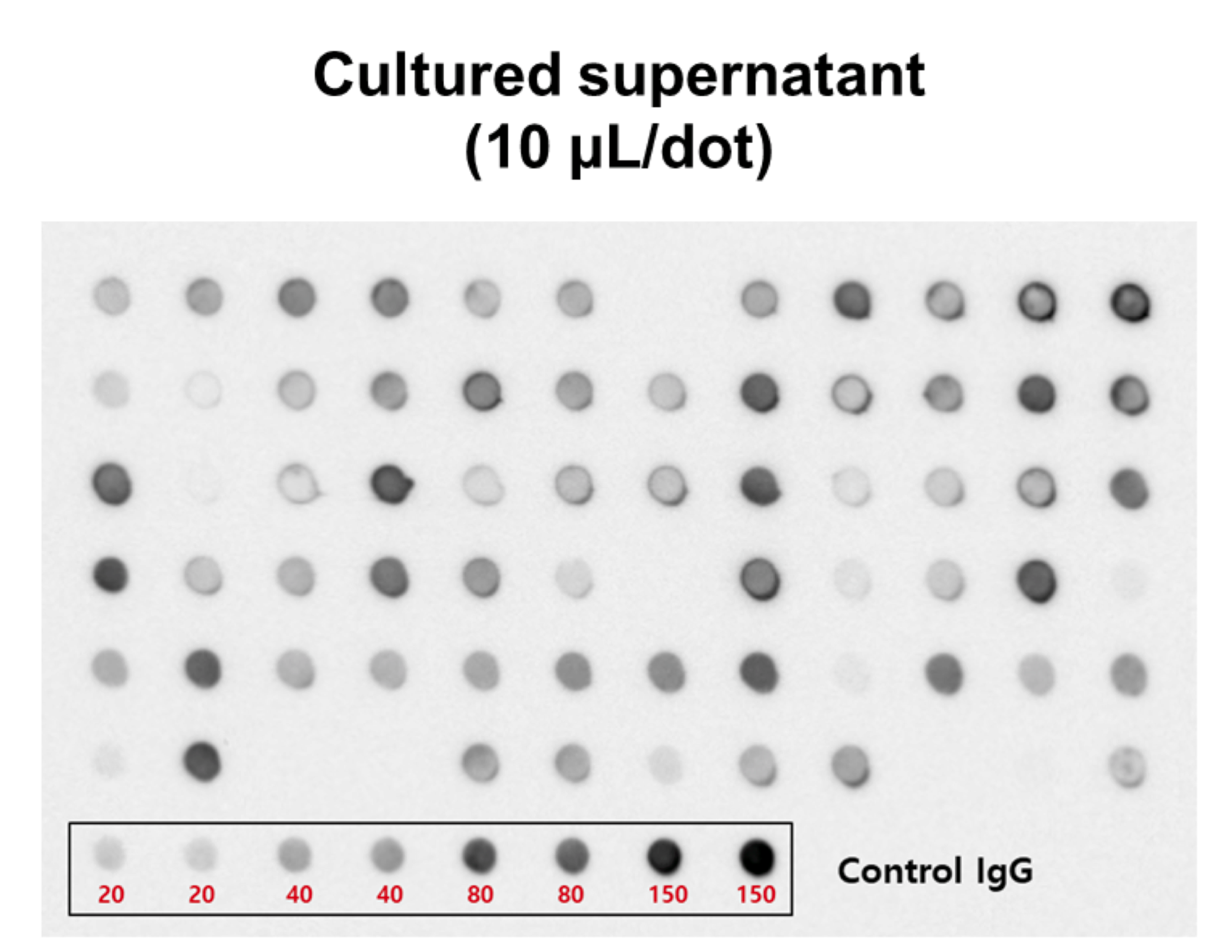

Supplement: Supplementary file 1 [file viruses-12-00684-s001.zip › Supplementary materials 300dp/Figure S2_rev2.tif]

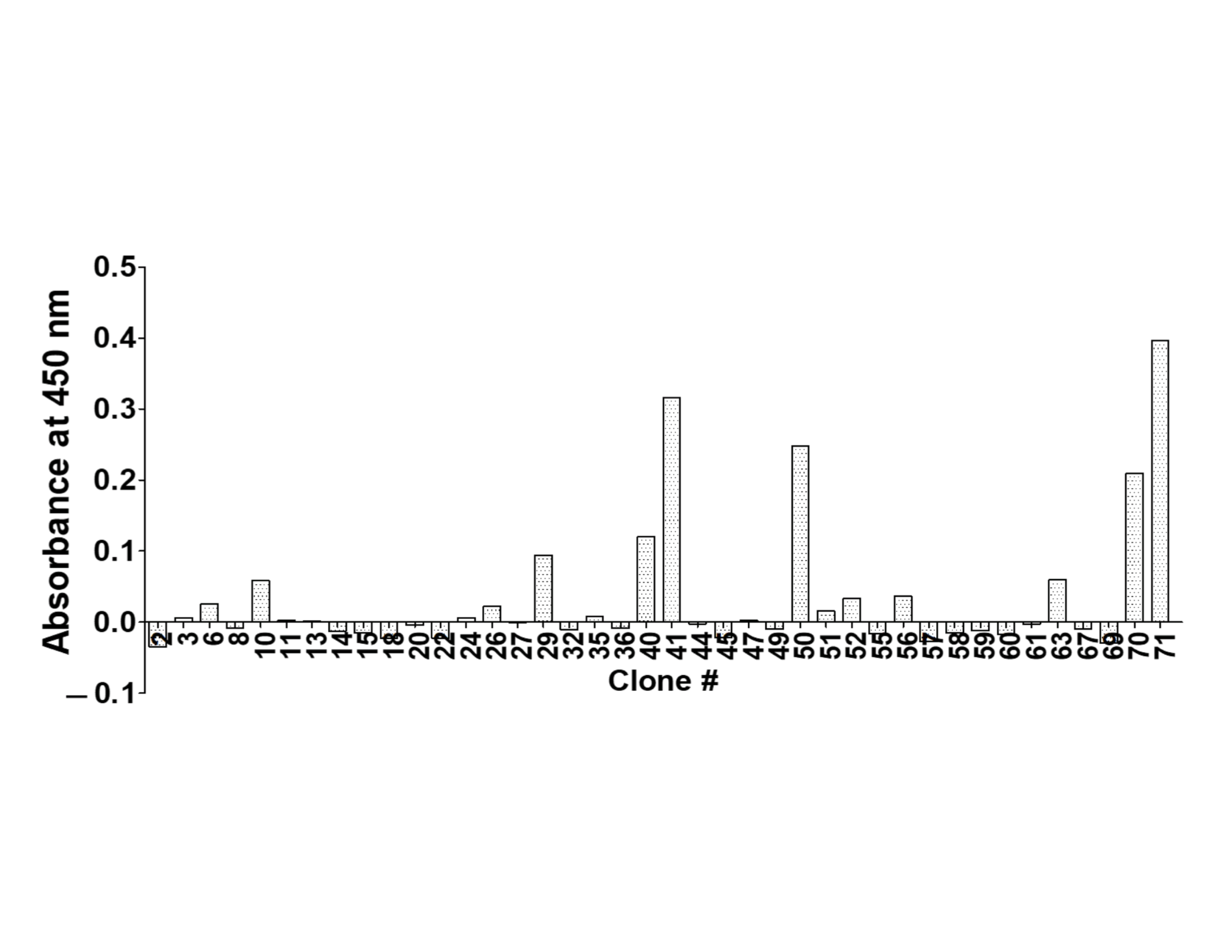

Supplement: Supplementary file 1 [file viruses-12-00684-s001.zip › Supplementary materials 300dp/Figure S3_rev2.tif]
